# Supplementary material for: A genome-wide association study based on the China Kadoorie Biobank identifies genetic associations between snoring and cardiometabolic traits
Source: Commun Biol. 2024 Mar 9;7:305. doi: 10.1038/s42003-024-05978-0 (PMC10924953; doi:10.1038/s42003-024-05978-0)
Supplement: Supplementary file 2 — Supplementary Information [file 42003_2024_5978_MOESM2_ESM.pdf]

(a)

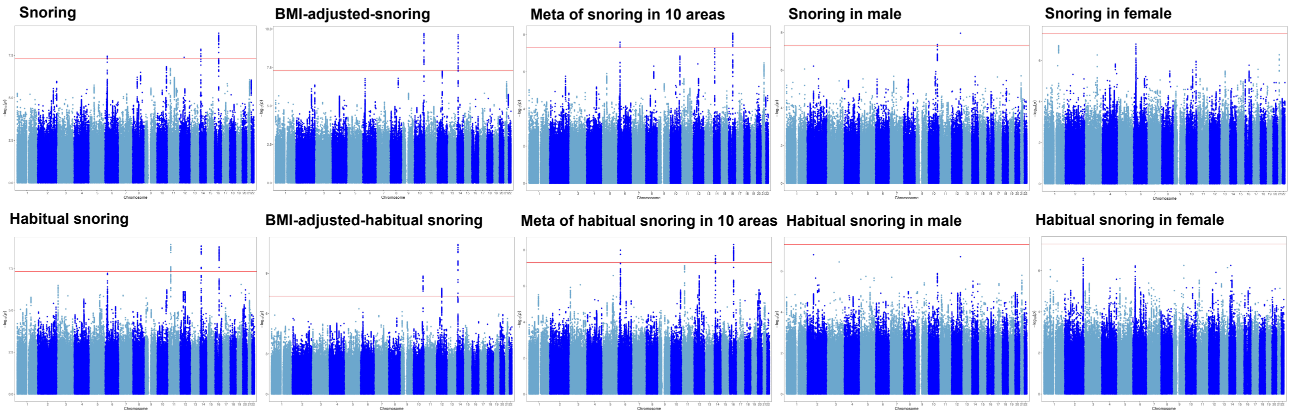

(b)

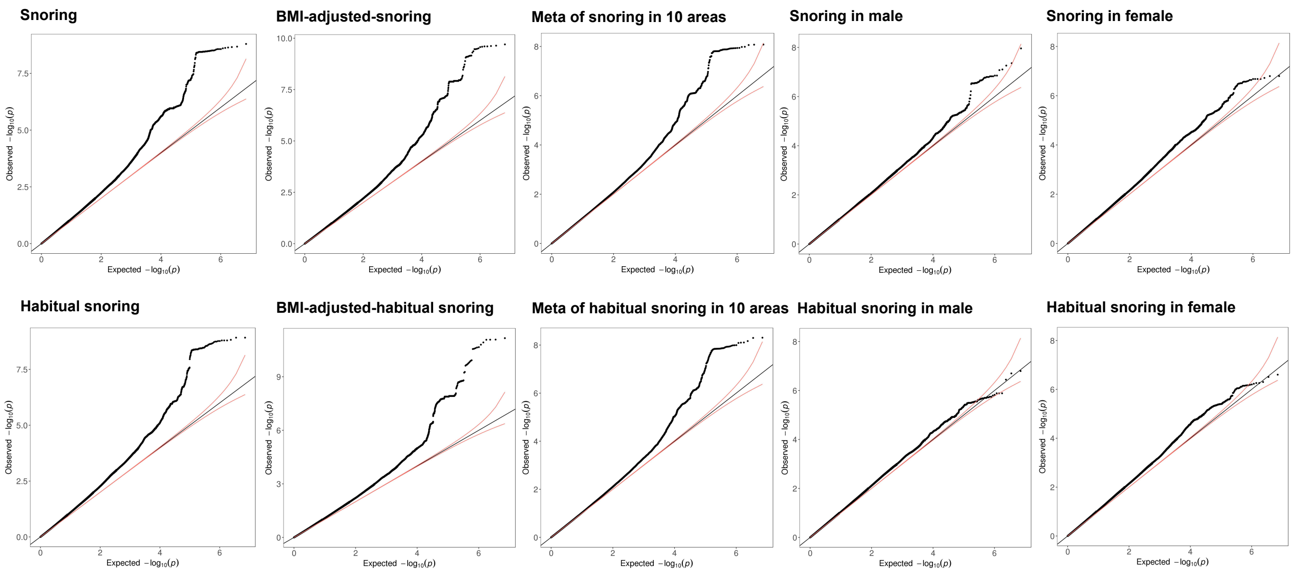

(c)

Traits		No. loci	λGC	LDSC intercept (SE)
Snoring	Main	4	1.097	1.015 (0.0075)
	BMI-adjusted	2	1.097	1.012 (0.0075)
	Meta-analysis of 10 study areas	2	1.025	0.946 (0.0067)
	Males	2	1.048	1.013 (0.0069)
	Females	0	1.097	1.017 (0.0078)
Habitual snoring	Main	3	1.147	1.011 (0.0072)
	BMI-adjusted	3	1.097	1.004 (0.0072)
	Meta-analysis of 10 study areas	3	1.017	0.939 (0.0064)
	Males	0	1.097	1.015 (0.0071)
	Females	0	1.097	1.019 (0.0075)

Supplementary Figure 1. GWAS and sensitivity GWAS results.

(a) Manhattan plots for GWAS of snoring, BMI-adjusted-snoring, Meta-analysis of snoring at ten study areas, snoring in males, snoring in females (on the panel above, from left to right); habitual snoring, BMI-adjusted-habitual snoring, Meta-analysis of habitual snoring at ten study areas, habitual snoring in male, habitual snoring in female (on the panel below, from left to right). For the Manhattan plots, X-axis denotes the genomic position (chromosomes 1-22), Y-axis denotes the $\log_{10}$(P-value) of association test. Genome-wide significance level ($P=5\times10^{-8}$) is denoted by red line.

(b) Quantile-quantile plots (QQ plots) for GWAS of the traits same as Manhattan plots.

(c) Summary table of the number of genomic risk loci (No.loci), λGC and the intercept of LD score (LDSC) regression in each GWAS.

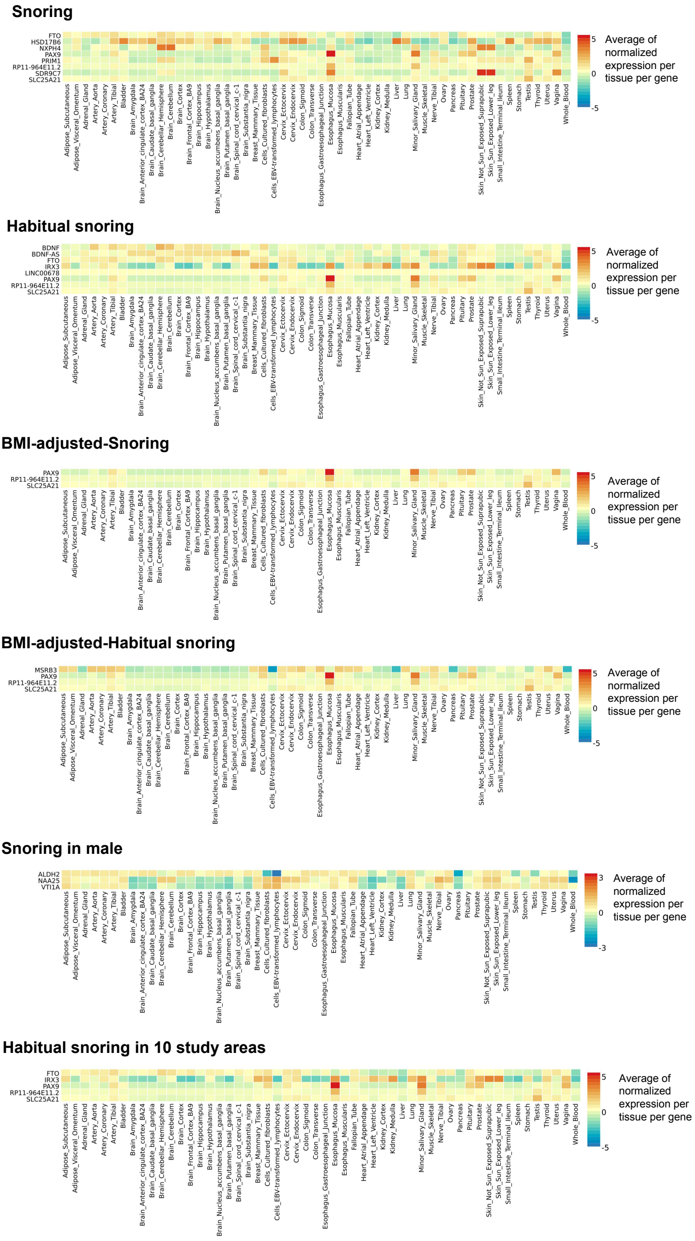

Supplementary Figure 2. Heatmaps of the expression in different tissues of the snoring genes. Notes: The gene expression heatmaps showed the average of normalized expression per tissue per gene (zero mean across samples) from Genotype-Tissue Expression, which allowed the comparison of gene expression across tissues within a gene. Only one gene for snoring at 10 study areas was not enough to perform the expression analysis. The source data could be found in Supplementary Data 7.

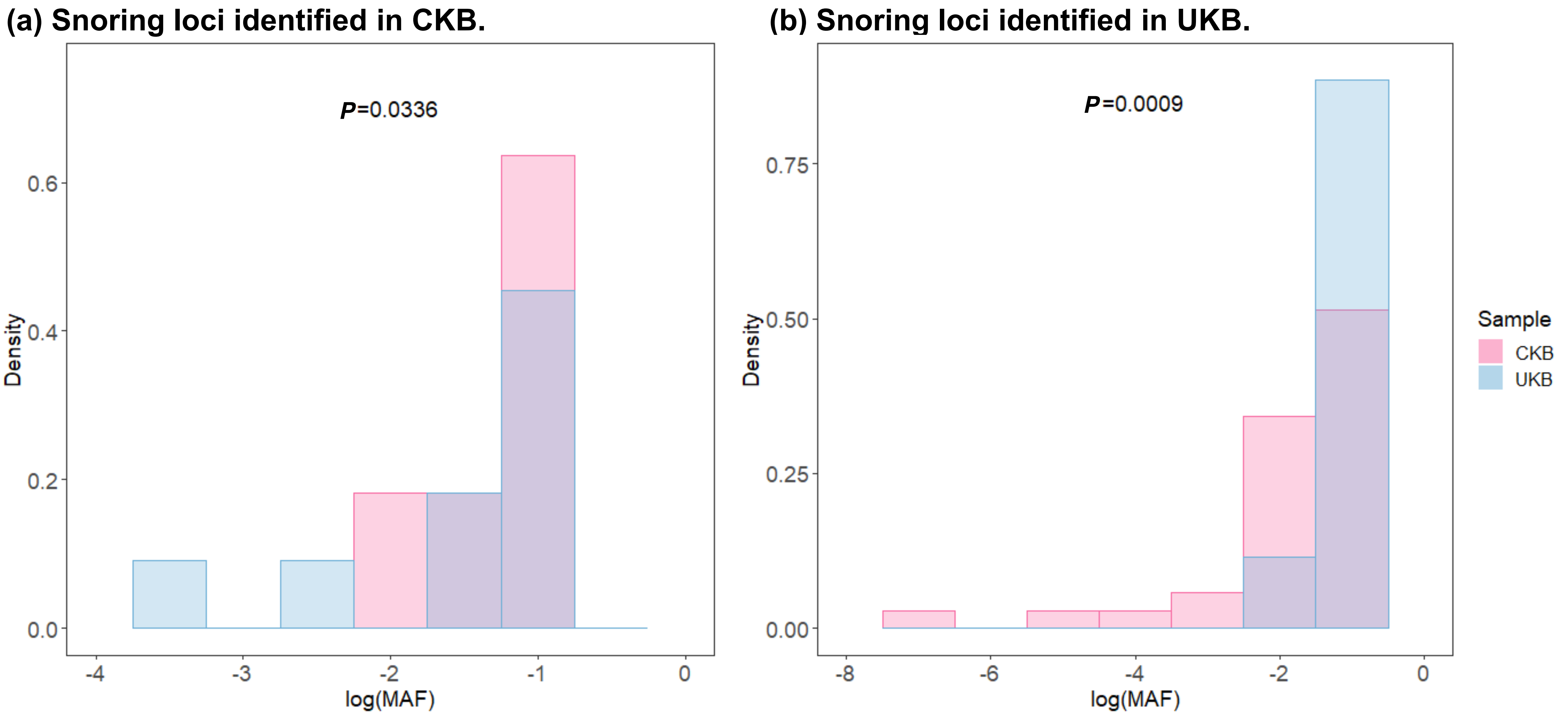

Supplementary Figure 3. Minor allele frequencies (MAF) comparison for snoring loci between CKB and UKB samples. Notes: CKB, China Kadoorie Biobank; UKB, UK Biobank. The present study compared MAFs between the CKB and UKB samples for the snoring loci identified in the CKB (panel a) and UKB (panel b) GWASs of snoring. Mann-Whitney U test *P* values were provided (two-sided test). The MAFs of the snoring loci in the two samples could be found in Supplementary Data 10.

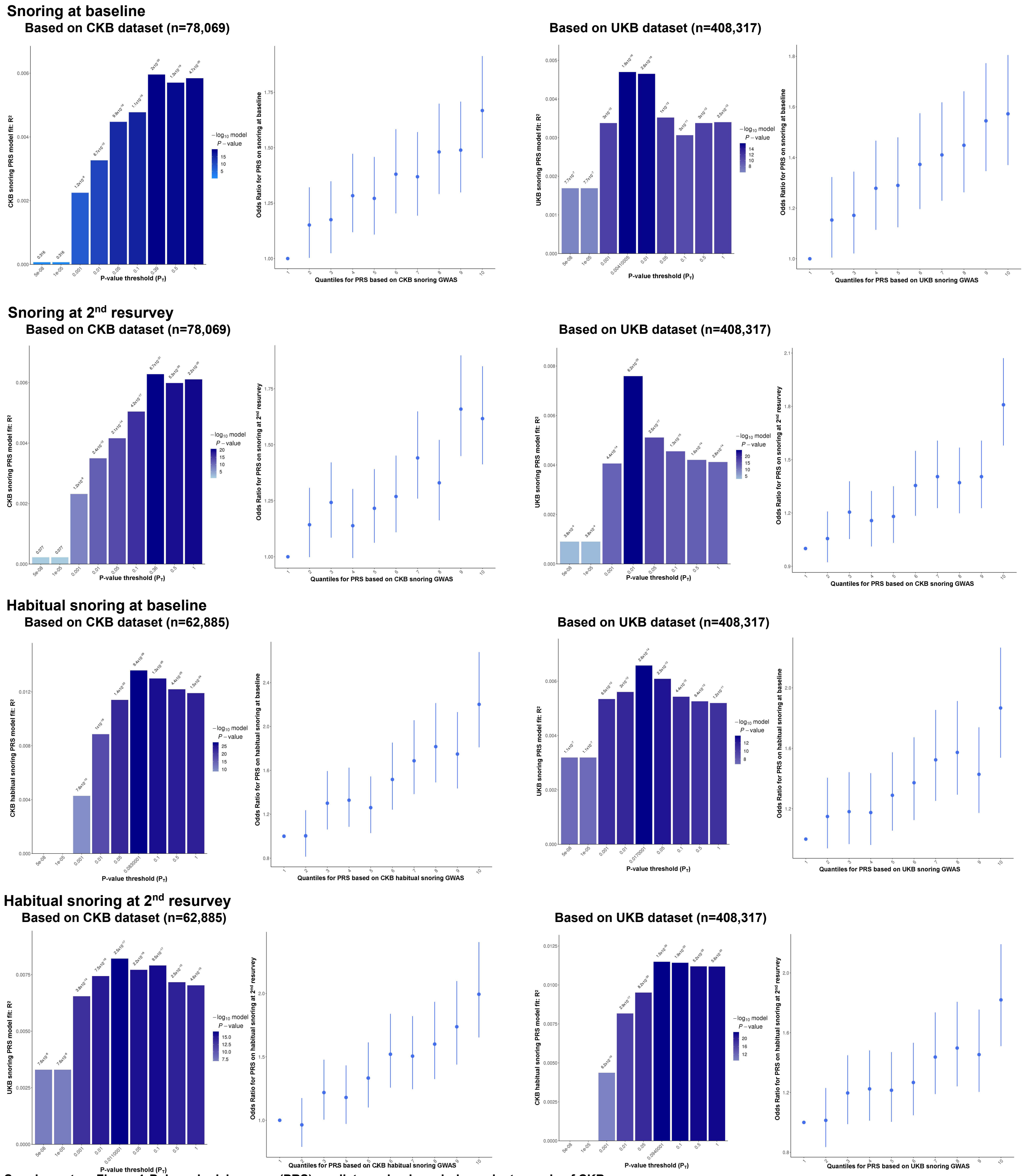

Supplementary Figure 4. Polygenic risk scores (PRS) predict snoring in an independent sample of CKB.

Notes: CKB, China Kadoorie Biobank; UKB, UK Biobank; PC, principal components. Bar plots (Row 1 and 3) showed the variance of snoring at baseline and 2nd resurvey, habitual snoring at baseline and 2nd resurvey (from top to bottom) in an independent sample of CKB ($n_{snoring}$=17,951, $n_{habitual\text{-}snoring}$=11,494), explained by PRS calculated from CKB discovery sample (row 1, $n_{snoring}$=78,069, $n_{habitual\ snoring}$=62,885) and UKB discovery sample (row 3, n=408,317). Full models for PRS construction were adjusted for age, age^2, sex, study areas, the first ten PCs, genotyping array, and baseline disease status; null models excluded PRS. The x-axis represented the P-value threshold used for variant inclusion, the y-axis represented the amount of variance explained by PRS (Nagelkerke R^2 of the change from null to full models). The exact *P*-value (Wald's test) is shown above each bar. Forest plots (Row 2 and 4) showed the odds ratios (95%CI) by decile of PRS for snoring from CKB (row 2) and UKB (row 4) (relative to the bottom decile=1) for the four snoring traits. The values of the odds ratios could be found in Supplementary Data 13.

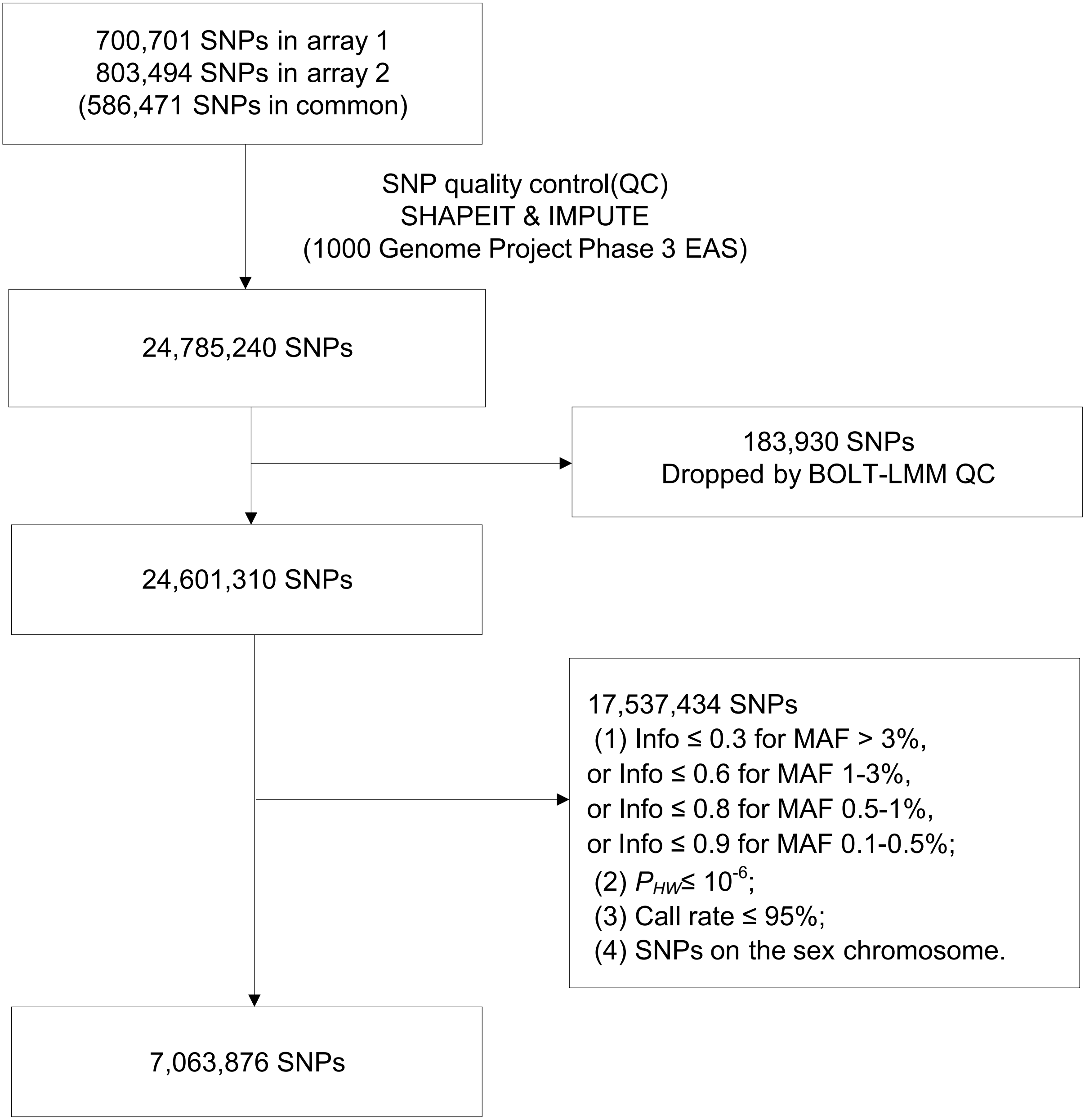

Supplementary Figure 5. A framework of SNPs selection

Notes: Single nucleotide polymorphisms; Info: information measure; MAF: minor allele frequency; P_{HW}: Hardy-Weinberg equilibrium deviations *P*.

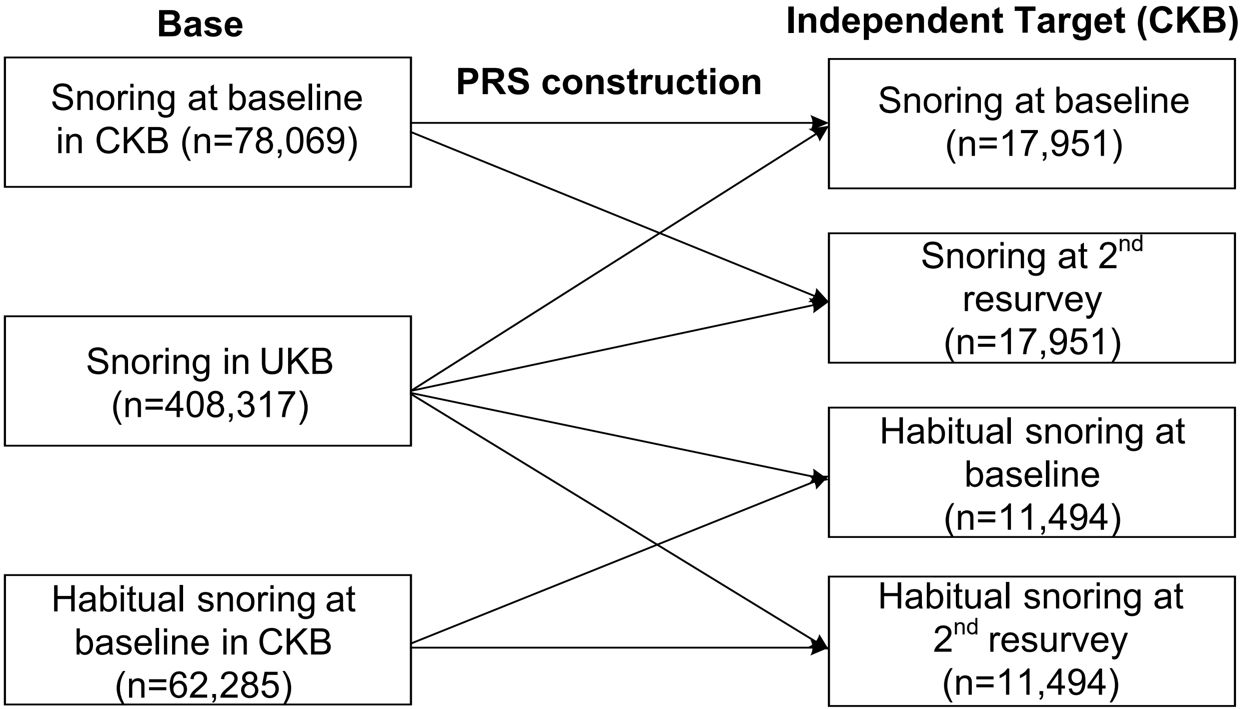

Supplementary Figure 6. A framework of PRS construction

Notes: PRS, polygenetic risk scores; CKB, China Kadoorie Biobank, UKB, UK Biobank.
