# Supplementary material for: A genome-wide association study based on the China Kadoorie Biobank identifies genetic associations between snoring and cardiometabolic traits
Source: Commun Biol. 2024 Mar 9;7:305. doi: 10.1038/s42003-024-05978-0 (PMC10924953; doi:10.1038/s42003-024-05978-0)
Supplement: Supplementary file 3 — Description of Additional Supplementary Files [file 42003_2024_5978_MOESM3_ESM.docx]

**Description of Additional Supplementary Files**

**File name:** Supplementary Data 1

**Description:** Snoring frequency among 10 study areas

**File name**: Supplementary Data 2

**Description:** Baseline characteristics of the CKB participants in the present study (N=100,626)

**File name:** Supplementary Data 3

**Description:** Genomic risk loci of the main and sensitivity GWAS of snoring

**File name:** Supplementary Data 4

**Description:** Novel loci for the GWAS of snoring in CKB

**File name:** Supplementary Data 5

**Description:** Annotation of snoring and habitual snoring GWAS

**File name:** Supplementary Data 6

**Description:** Results of eQTL mapping for snoring GWAS and the sensitivity analysis

**File name:** Supplementary Data 7

**Description:** Expression of snoring genes across 54 tissues from GTEx v8

**File name:** Supplementary Data 8

**Description:** Summary of mapped genes

**File name:** Supplementary Data 9

**Description**: Geneset enrichment analysis of snoring genes

**File name:** Supplementary Data 10

**Description:** Minor allele frequencies (MAF) comparison for snoring loci between EastAsian population of CKB and European population of UKB

**File name:** Supplementary Data 11

**Description:** Replication of CKB snoring SNPs in UKB snoring GWAS

**File name:** Supplementary Data 12

**Description:** Replication of UKB snoring SNPs in CKB snoring GWAS

**File name:** Supplementary Data 13

**Description**: Best PRSs predicted on the respective snoring traits

**File name:** Supplementary Data 14

**Description:** Results of PRS predict snoring or habitual snoring at baseline or 2nd resurvey in the independent target samples of CKB

**File name:** Supplementary Data 15

**Description:** Genetic correlations between snoring and cardiometabolic traits among the East Asians

**File name:** Supplementary Data 16

**Description:** Genetic correlations between snoring and cardiometabolic traits among the Europeans

**File name:** Supplementary Data 17

**Description:** Results of Outcome-related traits from PhenoScanner

**File name:** Supplementary Data 18

**Description:** F statistic of each SNPs on the corresponding exposure

**File name:** Supplementary Data 19

**Description:** Tests for MR analysis

**File name:** Supplementary Data 20

**Description:** Results of MR analysis of snoring with the cardiometabolic traits

**File name:** Supplementary Data 21

**Description:** GWAS of Cardiometabolic traits in the Biobank of Japan

**File name:** Supplementary Data 22

**Description:** GWAS of Snoring and Cardiometabolic traits in the UK Biobank
